# Establishment and Validation of a Novel Risk Score for Hepatocellular Carcinoma Based on Bile Acid and Bile Salt Metabolism-Related Genes

**DOI:** 10.3390/ijms24108597

**Published:** 2023-05-11

**Authors:** Qingmiao Shi, Xin Yuan, Chen Xue, Xinyu Gu, Lanjuan Li

**Affiliations:** State Key Laboratory for Diagnosis and Treatment of Infectious Diseases, National Clinical Research Center for Infectious Diseases, National Medical Center for Infectious Diseases, Collaborative Innovation Center for Diagnosis and Treatment of Infectious Diseases, The First Affiliated Hospital, Zhejiang University School of Medicine, Hangzhou 310003, China; shiqingmiao@zju.edu.cn (Q.S.); 12118235@zju.edu.cn (X.Y.); 11918229@zju.edu.cn (C.X.); 11918228@zju.edu.cn (X.G.)

**Keywords:** hepatocellular carcinoma, bile acid, risk model, prognosis, tumor microenvironment, immunotherapy

## Abstract

Liver cancer is a public disease burden with an increasing incidence rate globally. Bile acid and bile salt’s metabolic pathways participate in liver tumorigenesis and regulate the tumor microenvironment. However, there still remains a lack of systematic analysis of the genes related to bile acid and bile salt metabolic pathways in hepatocellular carcinoma (HCC). The mRNA expression data and clinical follow-up information of patients with HCC were obtained from public databases, including The Cancer Genome Atlas, Hepatocellular Carcinoma Database, Gene Expression Omnibus, and IMvigor210. The bile acid and bile salt metabolism-related genes were extracted from Molecular Signatures Database. Univariate Cox and logistic least absolute shrinkage and selection operator regression analyses were conducted to establish the risk model. Single sample gene set enrichment analysis, Estimation of STromal and Immune cells in MAlignant Tumour tissues using Expression data, and Tumor Immune Dysfunction and Exclusion were adopted to analyze immune status. The efficiency of the risk model was tested using a decision tree and a nomogram. We determined two molecular subtypes based on bile acid and bile salt metabolism-related genes, with the prognosis of the S1 subtype being markedly superior to the S2 subtype. Next, we established a risk model based on the differentially expressed genes between the two molecular subtypes. The high-risk and low-risk groups showed significant differences in the biological pathways, immune score, immunotherapy response, and drug susceptibility. Our results demonstrated the good predictive performance of the risk model in immunotherapy datasets and established that it could be an essential factor affecting the prognosis of HCC. In conclusion, we identified two molecular subtypes based on bile acid and bile salt metabolism-related genes. The risk model established in our study could effectively predict the prognosis of patients with HCC and their immunotherapeutic response, which may contribute to targeted immunotherapy in HCC.

## 1. Introduction

Liver cancer is a public health burden with the lowest overall survival rate among all other malignancies, and its incidence rate is increasing worldwide [1,2]. The population of newly confirmed clinical cases of liver cancer is estimated to increase by 55.0% per year between 2020 and 2040, with 1.4 million people possibly being diagnosed in 2040 [3]. China has the heaviest burden of liver cancer globally, accounting for 45.3% of the world’s diagnosed cases and 47.1% of liver cancer-associated deaths in 2020. As the most ubiquitous type of primary liver cancer, hepatocellular carcinoma (HCC) accounts for approximately 90% of all cases [4]. Although most patients with HCC receive systematic management during their disease course, the efficacy is still unsatisfactory. Given the limitation of HCC treatment strategies, establishing a novel model to predict the risk of HCC is necessary.

Bile acids are soluble derivatives produced during the catabolism of cholesterol in the liver via the classical pathway initiated by cholesterol 7-hydroxylase and the non-classical pathway initiated by sterol 27A hydroxylase. Primary bile acids in the liver are released into the intestine through the gallbladder, where some of them are converted into secondary bile acids by gut-colonizing bacteria [5,6,7]. Most of the intestinal bile acids undergo enterohepatic circulation, where they are reabsorbed into the liver [8]. Studies have confirmed that bile acids and their metabolites can regulate the body’s metabolism and energy homeostasis by activating dedicated receptors such as farnesoid X receptor and Takeda G-protein-coupled receptor-5 [9]. Bile acids-farnesoid X receptor and bile acids-Takeda G-protein-coupled receptor-5 signaling pathways are closely associated with metabolic disorders, including Type 2 diabetes mellitus, non-alcoholic steatohepatitis, obesity, and atherosclerosis [10,11].

Decades of evidence have indicated that bile acids can regulate the onset and progression of HCC. Dawang Zhou and his colleagues found that several patients with liver cancer had increased serum bile acid levels and inactivation of the hepatocyte Hippo signaling pathway [12]. They further showed that fibroblast growth factor 15 could activate the Hippo signaling pathway by binding to its receptor, fibroblast growth factor receptor 4, and thereby inhibiting bile acid synthesis and liver tumorigenesis. Ma et al. reported that intestinal commensal bacteria could regulate the expression of chemokine ligand 16 in hepatic sinusoidal endothelial cells through bile acids as messengers, which promotes the accumulation of hepatic chemokine receptor 6^+^ natural killer T cells, thereby achieving an anti-tumor effect in the liver [13]. Another study revealed that the downregulation of Sirtuin 5 increased the production of bile acids [14]. Bile acids activate their nuclear receptors and induce the polarization of M2-like macrophages, leading to an immunosuppressive microenvironment that facilitates the immune escape of hepatoma cells. Given that bile acid homeostasis can regulate the tumor microenvironment and the progression of HCC, a systematic analysis of the bile acid and bile salt metabolism-related genes may be beneficial for identifying prognostic molecular markers and developing potential strategies for the early diagnosis and management of patients with HCC.

In this study, bile acid and bile salt metabolism-related genes were utilized to determine two molecular subtypes in patients with HCC through consensus clustering analysis. Next, we identified risk factors based on differentially expressed genes (DEGs) between the two molecular subtypes. In addition, we established a clinical risk model with good efficiency in predicting the patients’ survival. We also evaluated the biological pathways, immune score, immunotherapy response, and drug sensitivity in different risk subgroups. Finally, we established a survival decision tree and nomogram by combining the risk score and clinicopathological features to quantify the risk assessment and probability of survival.

## 2. Results

### 2.1. Determination of Molecular Subtypes Based on Bile Acid and Bile Salt Metabolism-Related Genes

**Figure 1:**
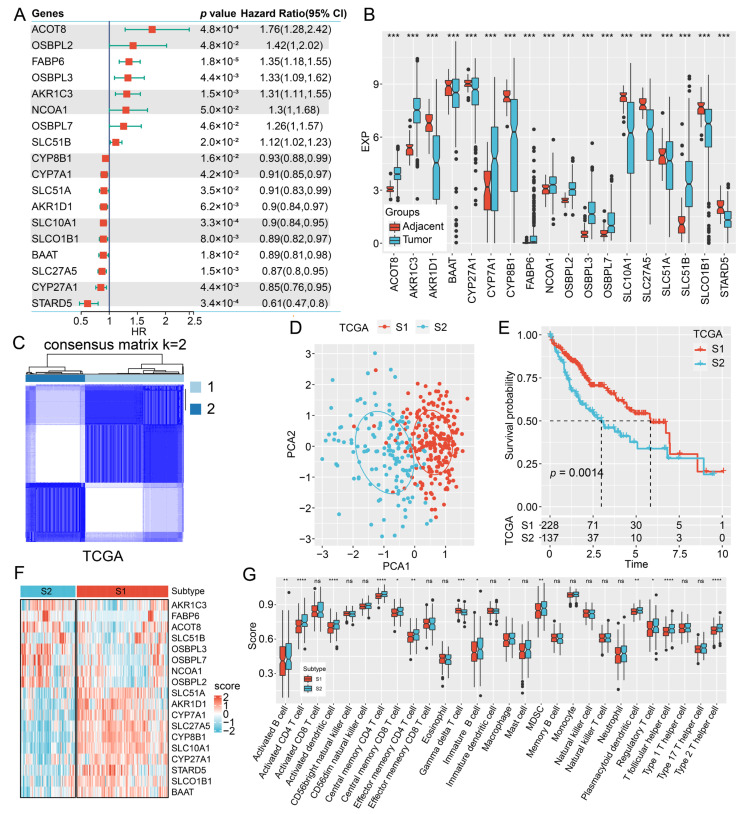
Determination of molecular subtypes based on bile acid and bile salt metabolism-related genes in the TCGA dataset. (**A**) Univariate Cox analysis of 18 bile acid and bile salt metabolism-related genes. (**B**) Differences in the mRNA expression levels of 18 genes in HCC and the adjacent tissues. (**C**) Consensus matrix heatmap based on K = 2. (**D**) Principal component analysis of the TCGA samples based on the two molecular subtypes. (**E**) Survival analysis of the two molecular subtypes. (**F**) Heatmap of the mRNA expression levels of 18 prognostic genes in the two subtypes. (**G**) The immune score of two molecular subtypes through the ssGSEA method. *, *p* < 0.05; **, *p* < 0.01; ***, *p* < 0.001; ****, *p* < 0.0001; ns, non-significant.

We obtained 45 genes related to bile acid and bile salt metabolism pathways from the Molecular Signatures Database (MSigDB). To explore the function of bile acid and bile salt metabolism in predicting the outcome of HCC, we conducted a univariate Cox regression analysis on the mRNA expression levels of the obtained genes based on The Cancer Genome Atlas (TCGA) dataset. The results indicated a significant association between the 18 genes and the prognosis of HCC. Among them, eight genes, including ACOT8, OSBPL2, FABP6, OSBPL3, AKR1C3, NCOA1, OSBPL7, and SLC51B, were risk factors. In contrast, 10 genes, namely CYP8B1, CYP7A1, SLC51A, AKR1D1, SLC10A1, SLCO1B1, BAAT, SLC27A5, CYP27A1, and STARD5 were protective factors (Figure 1A). We then evaluated the discrepancies in the mRNA expression levels of these 18 genes in HCC and the adjacent tissues. All 18 genes exhibited a significant change in their expression profiles. Among these, the expression levels of nine were upregulated and the other nine were downregulated in HCC (Figure 1B).

**Figure 2:**
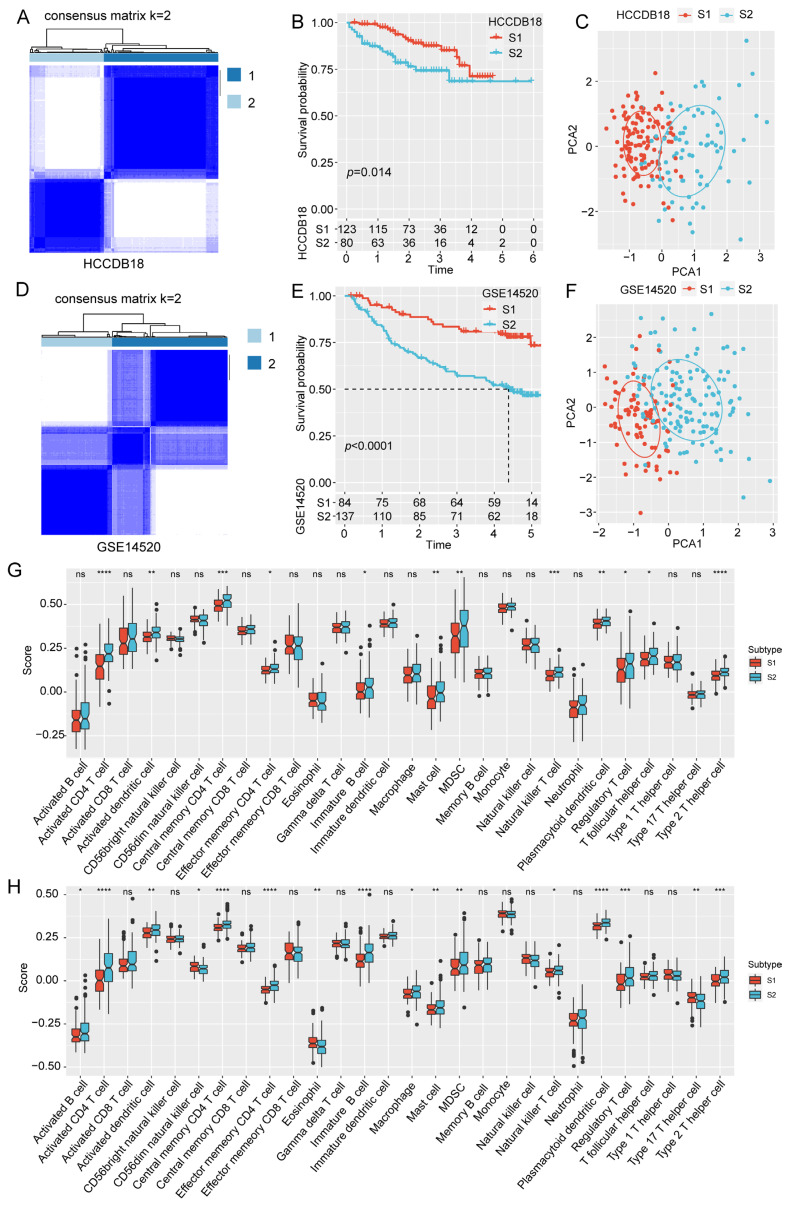
Validation of the two molecular subtypes in the HCCDB18 and GSE14520 datasets. (**A**) Consensus matrix heatmap, (**B**) survival curve, and (**C**) Principal component analysis of the two molecular subtypes in the HCCDB18 dataset. (**D**) Consensus matrix heatmap, (**E**) survival curve, and (**F**) Principal component analysis of the two molecular subtypes in GSE14520 datasets. (**G**) The immune score of the two molecular subtypes in the HCCDB18 dataset. (**H**) The immune score of the two molecular subtypes in the GSE14520 dataset. *, *p* < 0.05; **, *p* < 0.01; ***, *p* < 0.001; ****, *p* < 0.0001; ns, non-significant.

Based on the expression profile of 18 bile acid and bile salt metabolism genes, we performed a consensus clustering analysis on the TCGA cohort. The consensus matrix heatmap presented two different molecular subtypes when K was selected as 2 (Figure 1C). Principal component analysis of the TCGA samples showed that the two subtypes could be clearly distinguished, which proved the rationality of the classification (Figure 1D). The survival analysis of the two molecular subtypes suggested that the prognosis of S1 subtype was markedly superior to that of S2 subtype (Figure 1E). The heatmap showed the mRNA expression of 18 prognostic genes of the two subtypes (Figure 1F). Consistent results were observed in the Hepatocellular Carcinoma Database 18 (HCCDB18) (Figure 2A–C) and Gene Expression Omnibus (GEO) Series 14520 (GSE14520) (Figure 2D–F) databases.

Furthermore, the immune infiltration status was investigated in the distinct molecular subtypes. We utilized single sample gene set enrichment analysis (ssGSEA) to assess the immune status and differences among the subtypes according to the immune cell genes reported by previous studies [15,16]. Most immune cells in the TCGA dataset, such as activated B cells, activated dendritic cells, and Type 2 T helper cells, exhibited a high expression trend in the S2 subtype (Figure 1G). Similar results were obtained in the HCCDB18 (Figure 2G) and GSE14520 datasets (Figure 2H). These results suggest that bile acid and bile salt metabolism-related genes are closely associated with the prognosis of HCC. Moreover, we determined two molecular subtypes based on the 18 prognosis genes, with different prognoses and degree of immune infiltration.

### 2.2. Identification of Risk Factors Based on the DEGs between the Two Molecular Subtypes

**Figure 3:**
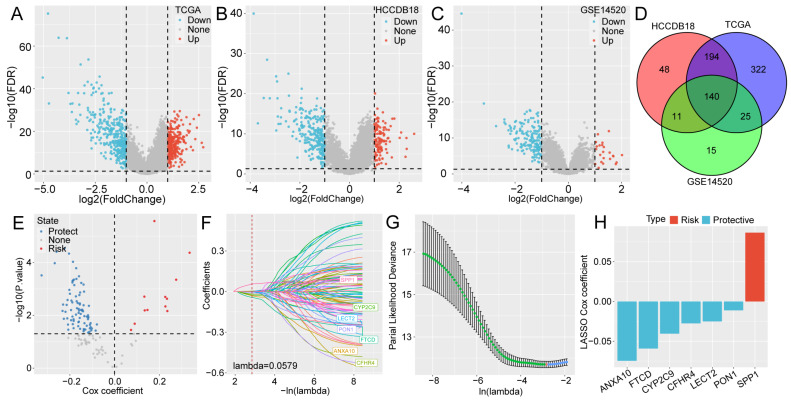
Risk factors based on the DEGs between the two molecular subtypes. (**A**–**C**) Volcano plots of the DEGs between the two subtypes in the (**A**) TCGA, (**B**) HCCDB18, and (**C**) GSE14520 datasets. (**D**) Venn diagram of the DEGs identified in the three datasets. (**E**) Univariate Cox regression analysis of 140 common DEGs in the TCGA dataset. (**F**) The trajectory of each independent variable as lambda changed. (**G**) Confidence intervals for different numerical values of lambda. (**H**) LASSO Cox coefficients of the seven prognostic genes.

The DEGs were identified between the two subtypes of TCGA (Figure 3A), HCCDB18 (Figure 3B), and GSE14520 (Figure 3C) datasets. We identified 140 common DEGs from the intersection of the DEGs in the three databases, as shown in the Venn diagram (Figure 3D). Then, we performed univariate Cox regression analysis on these 140 common DEGs in the TCGA dataset and found 95 genes with a greater prognostic value, including 12 genes with risk effect and 83 genes with a protective effect (Figure 3E). Additionally, logistic least absolute shrinkage and selection operator (LASSO) regression was performed to diminish the variables, and its coefficient trajectory diagram is displayed in Figure 3F. We found that the optimal model was determined when lambda was 0.0579 (Figure 3G). Consequently, seven genes were established as subsequent target genes, namely ANXA10, FTCD, CYP2C9, CFHR4, LECT2, PON1, and SPP1. The LASSO Cox coefficient indicated that SPP1 was a risk factor, while ANXA10, FTCD, CYP2C9, CFHR4, LECT2, and PON1 were protective factors (Figure 3H). The molecular mechanism of the seven genes involved in the progression of HCC deserves further exploration.

### 2.3. Establishment and Verification of Risk Model

**Figure 4:**
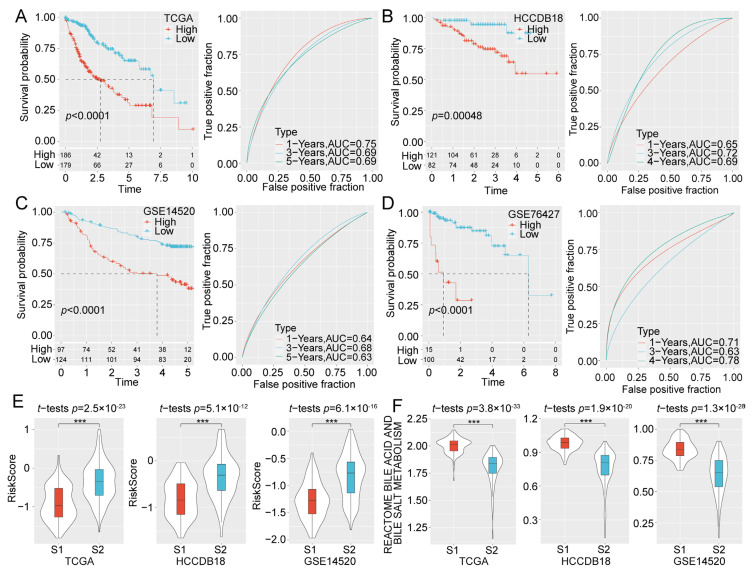
Establishment and validation of clinical risk model. KM survival and ROC curves based on RiskScore in the (**A**) TCGA, (**B**) HCCDB18, (**C**) GSE14520, and (**D**) GSE76427 datasets. (**E**) Differences of RiskScore between the two molecular subtypes in the TCGA, HCCDB18, and GSE14520 datasets. (**F**) Differences in bile acid and bile salt metabolism scores between the two molecular subtypes in the TCGA, HCCDB18, and GSE14520 datasets. ***, *p* < 0.001.

We assessed the RiskScore of each sample according to the formula and classified the samples as high-risk and low-risk groups. Kaplan-Meier curves showed that the survival probability of the low-risk group was significantly preferable to that of the high-risk group (Figure 4A). The receiver operator characteristic (ROC) curves suggested that the risk model had good predictive efficacy (Figure 4A). The robustness of the risk model was then verified in the HCC datasets HCCDB18 (Figure 4B), GSE14520 (Figure 4C), and GSE76427 (Figure 4D). We observed similar results in the validation cohorts demonstrating the significantly lower survival probability of high-risk group compared with the low-risk group. Overall, our findings suggested that the risk model was reliable in predicting patient survival.

Moreover, the differences in RiskScore and bile acid and bile salt metabolism scores in the two molecular subtypes were compared in TCGA, HCCDB18, and GSE14520 datasets. We found a higher RiskScore for the S1 subtype with the worst prognosis (Figure 4E). In contrast, the bile acid and bile salt metabolism scores were observably lower in S2 but higher in S1 (Figure 4F).

**Figure 5:**
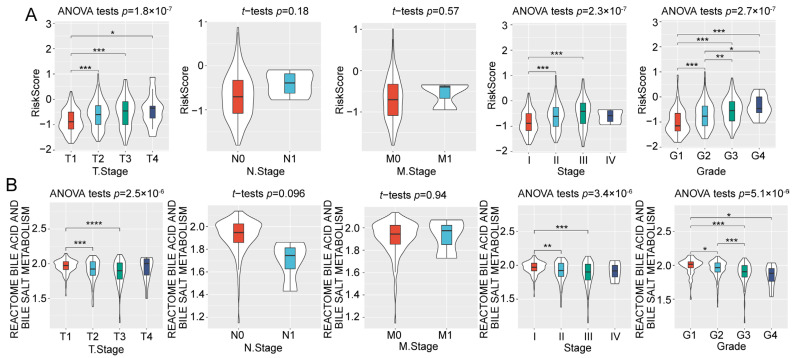
The performance of RiskScore and bile acid and bile salt metabolism scores in distinguishing clinicopathological features. (**A**) The difference of RiskScore in T Stage, N Stage, M Stage, Stage, and Grade. (**B**) The difference of bile acid and bile salt metabolism scores in T Stage, N Stage, M Stage, Stage, and Grade. *, *p* < 0.05; **, *p* < 0.01; ***, *p* < 0.001; ****, *p* < 0.0001.

Furthermore, we found that the RiskScore differed among the clinicopathological characteristics, including the T Stage, Stage, and Grade (Figure 5A). Notably, the RiskScore was significantly higher in tumors with a higher Grade, indicating its potential utility as a predictor of HCC grade. We also compared the differences between the clinicopathological features in the scores for bile acid and bile salt metabolism. The results also showed prominent differences in the scores for bile acid and bile salt metabolism and clinicopathological features, including T Stage, Stage, and Grade (Figure 5B).

### 2.4. Analysis of Biological Pathways and Immune Scores in Different Risk Subgroups

**Figure 6:**
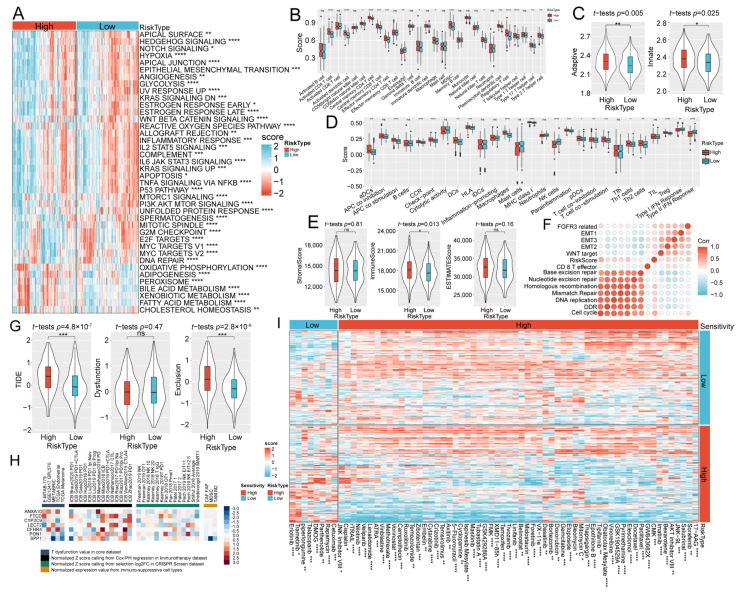
Analysis of pathways, immune scores, immunotherapy responses, and drug sensitivity in different risk subgroups. (**A**) Heatmap of the signaling pathways. (**B**) The immune score of the high-risk and low-risk groups. (**C**) Scores of adaptive and innate immunities in the two risk subgroups. (**D**,**E**) Overall immunity score of the TCGA dataset derived via the ESTIMATE method. (**F**) Correlation analysis of the RiskScore and the 13 marker genes of human-related pathways. (**G**) TIDE score of the two different risk subgroups. (**H**) The relationship between the expression of seven prognostic genes and several immunotherapeutic-related features based on the TIDE algorithm. (**I**) Sensitivity of traditional chemotherapeutic drugs in the high-risk and low-risk groups. *, *p* < 0.05; **, *p* < 0.01; ***, *p* < 0.001; ****, *p* < 0.0001; ns, non-significant.

To further elucidate the biological functions enriched in different risk subgroups, we performed ssGSEA on the TCGA cohort. We identified 40 biological pathways that differed significantly in the two risk subgroups. Pathways, such as oxidative phosphorylation, adipogenesis, peroxisome, bile acid metabolism, xenobiotic metabolism, fatty acid metabolism, and cholesterol homeostasis pathway, were enriched in the high-risk group (Figure 6A). Other pathways, such as the Hedgehog signaling and KI3K/AKT/MTOR signaling, were enriched in the low-risk group.

Furthermore, we applied the ssGSEA method to assess the immune infiltration state of the two risk subgroups. The results demonstrated that the high-risk group had a higher abundance of most immune cells, such as activated CD4+ T cells, myeloid-derived suppressor cells (MDSC), and regulatory T cells, than the low-risk group. (Figure 6B). The high-risk group also exhibited higher scores for adaptive immunity and innate immunity (Figure 6C) and higher overall immune scores as evaluated by Estimation of STromal and Immune cells in MAlignant Tumour tissues using Expression data (ESTIMATE) (Figure 6D,E). Additionally, we used 13 marker genes of human-related pathways obtained from a previous study [17] to calculate their scores by ssGSEA. We found that the RiskScore was positively associated with FGFR3-related, EMT3, EMT2, EMT1, Mismatch Repair, and other pathways (Figure 6F). A previous study found that FGFR3 was abnormally upregulated in HCC, and mis-splicing of FGFR3 mRNA significantly promoted the malignant characteristics of HCC [18]. Another study revealed that the EMT induced by transforming growth factor β in HCC contributed to cell migration and invasion [19]. Further studies are needed to better link the clinical management of HCC to relevant biomarkers and targeted therapies.

### 2.5. Evaluation of Immunotherapeutic Response and Drug Sensitivity

Furthermore, we used the Tumor Immune Dysfunction and Exclusion (TIDE) algorithm to evaluate the potential clinical response to immunotherapy in the high-risk and low-risk groups. The higher TIDE score was positively associated with immune escape. We found a lower TIDE score in the low-risk group than in the high-risk group (Figure 6G), suggesting that the low-risk group had a greater chance of gaining an advantage from the immunity treatment. In terms of the tumor immune dysfunction and the immune clearance process, the low-risk group exhibited a decreased T cell exclusion score and an increased T cell dysfunction score.

To uncover the potential gene targets in combined immunotherapy, on the basis of the TIDE algorithm, we assessed the association between the expression of seven genes and several immunotherapy-related features, including T cell dysfunction, the outcome of the immune checkpoint blockade response, phenotypes of CRISPR screening, and immuno-suppressive cell types (Figure 6H). Our findings suggested that the high expression of ANXA10 was positively associated with T-cell dysfunction. In cell types that facilitated T cell rejection, the expression levels of SPP1 in MDSC cells and TAM M2 cells were significantly decreased. Next, we evaluated the sensitivity of traditional chemotherapeutic drugs and compared the differences of 68 drugs in the risk subgroups (Figure 6I). Among them, 60 drugs, including Sorafenib, Salubrinal, and Bexarotene, were sensitive to the high-risk group, while only 8 drugs were sensitive to the low-risk group, including Cetuximab, Rapamycin, Parthenolide, DMOG, Talazoparib, Piperlongumine, Trametinib, and Erlotinib. Our findings might provide a basis for drug selection for immune treatment of HCC.

### 2.6. Predictive Performance of Prognostic Models in Immunotherapy Datasets

**Figure 7:**
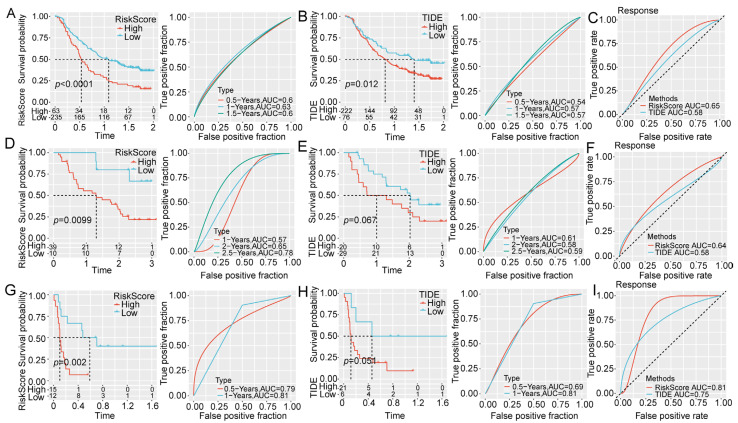
Predictive performance of prognostic models in the immunotherapeutic datasets. Kaplan-Meier survival curve and ROC curve of RiskScore in the (**A**) IMvigor210, (**D**) GSE91061, and (**G**) GSE135222 datasets. Kaplan-Meier survival curve and ROC curve of TIDE score in the (**B**) IMvigor210, (**E**) GSE91061, and (**H**) GSE135222 datasets. The ROC curve of RiskScore and TIDE for predicting the immunotherapy response in the (**C**) IMvigor210, (**F**) GSE91061, and (**I**) GSE135222 datasets.

The cohorts included in the HCC datasets, IMvigor210, GSE91061, and GSE135222, were all patients who had undergone immunotherapy. The RiskScore and TIDE score were obtained from these data to evaluate the immunotherapeutic response. The survival curve demonstrated distinct RiskScores between the survival possibilities of the different subgroups in the three datasets (Figure 7A,D,G). However, the TIDE score exhibited good predictive performance only in the cohort of the IMvigor210 dataset (Figure 7B) but failed to distinguish the survival in the cohort of the GSE91061 (Figure 7E) and GSE135222 datasets (Figure 7H). Overall, the RiskScore was superior to the TIDE score in predicting the treatment response (Figure 7C,F,I).

### 2.7. RiskScore Serves as a Valuable Prognostic Factor for Patients with HCC

**Figure 8:**
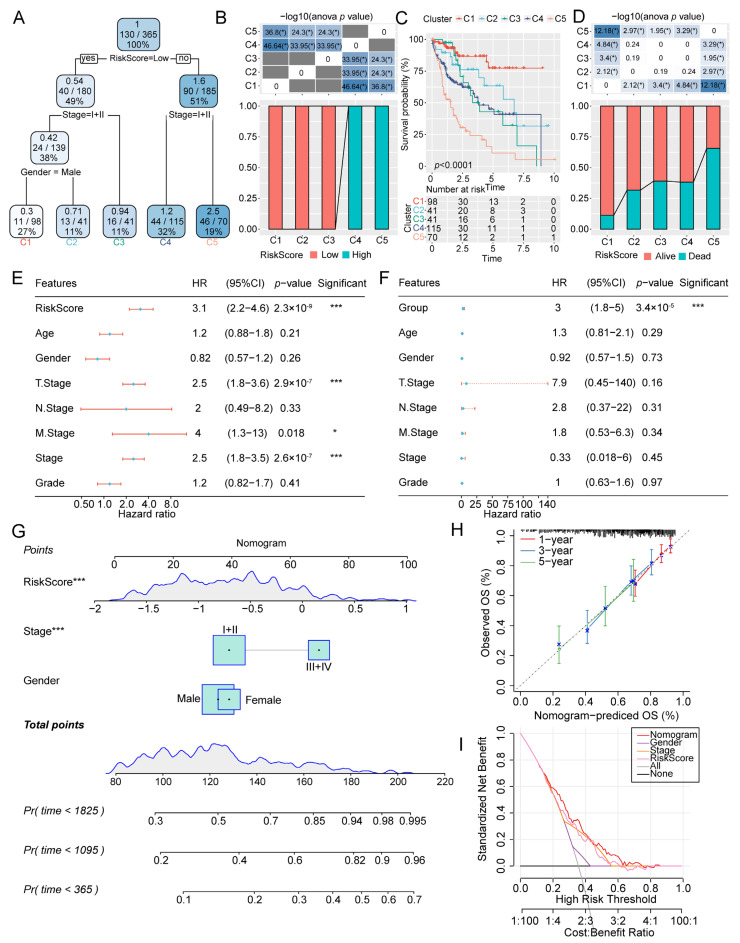
Improving the prognostic model and survival prediction by combining RiskScore with clinicopathological features. (**A**) Decision tree including Group, Stage, and Gender. (**B**) Survival analysis of the five risk subgroups. (**C**) Different risks of patients from the five subgroups. (**D**) Different survival statuses of patients from the five subgroups. (**E**) Univariate and (**F**) multivariate Cox regression analyses of RiskScore and clinicopathological features. (**G**) Nomogram including RiskScore, Stage, and Gender. (**H**) Calibration curves of the nomogram of the three points in 1-year, 3-year, and 5-year. (**I**) Decision curve of the nomogram. *, *p* < 0.05; ***, *p* < 0.001.

Next, we constructed a survival decision tree to optimize risk stratification in the TCGA cohort and found that the Group, Stage, and Gender constituted the final decision-making scheme. We identified five risk clusters, with Group and T Stage being the most potent parameters (Figure 8A). Patients in the risk clusters C1, C2, and C3 were low-RiskScore, whereas C4 and C5 were high-RiskScore patients (Figure 8C). The five risk subgroups demonstrated significant differences in their survival rates (Figure 8B). Additionally, we also uncovered differences in the survival outcome in the five risk subgroups (Figure 8D). Furthermore, univariate (Figure 8E) and multivariate (Figure 8F) Cox regression analyses indicated that RiskScore was the most remarkable factor affecting prognosis.

Moreover, we established a nomogram by combining RiskScore, Stage, and Gender (Figure 8G) and found that RiskScore had the most significant influence on survival prediction. Furthermore, we observed that the prediction calibration curves of 1, 3, and 5 years were close to the standard curve, indicating that the nomogram had excellent performance (Figure 8H). In addition, the decision curve also showed that the nomogram and RiskScore possessed superior survival prediction ability (Figure 8I). Therefore, our findings suggest that RiskScore could potentially serve as a valuable prognostic factor for patients with HCC.

## 3. Discussion

The liver plays a crucial role in various physiological functions, such as metabolism, immune regulation, lipid and cholesterol homeostasis, and the synthesis of coagulation factors [20]. Bile acids are initially derived from the metabolism of cholesterol in hepatocytes [21]. The efficient enterohepatic circulation of bile acids between the liver and small intestine maintains the systemic homeostasis of bile acids, participates in the digestion and absorption of various nutrients, and affects the structure and function of the gut microbiota [22]. Dysregulation of bile acid metabolism is involved in tumor progression, especially in gastrointestinal cancer [23,24,25,26]. Numerous studies have revealed that hydrophobic bile acids such as lithocholic acid, deoxycholic acid, and chenodeoxycholic acid are significant promoters of liver cancer [27,28]. Moreover, the decrease in farnesoid X receptor signaling during liver inflammation leads to a reduction of hepatic bile acid transporters such as BSEP, OSTα/β, MRP2, MDR2-3, and NTCP, leading to an increase in the concentration of bile acid and persistent inflammation in the liver, which could facilitate the occurrence of HCC [9,29].

This study used univariate Cox regression analysis to investigate the association of 18 genes related to bile acid and bile salt metabolism with HCC prognosis. All 18 genes’ expression levels significantly differed between HCC and adjacent tissues, indicating their involvement in carcinogenesis. Furthermore, we identified two molecular subtypes of HCC based on the expression profiles of these prognostic genes, which exhibited different prognoses and levels of immune infiltration. We observed that most immune cells showed a tendency of high expression in the S2 subtype with poor prognosis, indicating that the heterogeneity of immune infiltration in HCC may act as a prognostic indicator. Therefore, the risk prediction based on the metabolic pathways of bile acid and bile salt may provide a more effective prognostic estimation for HCC.

A previous study reported by NEJM investigated the gene-expression profiles of HCC tissues, but failed to detect significant gene expressions correlated with either tumor recurrence or survival [30]. In spite of this, the further assessment of the gene-expression profiles of non-tumor liver tissues identified that the aggregate survival-correlated signature contained 186 genes, and functional annotation of the survival signature by GSEA found that the “bile acid biosynthesis” gene set was correlated with good survival. In our study, we development a novel risk score for HCC based on bile acid and bile salt metabolism-related genes, and the risk model could effectively predict the prognosis of patients with HCC. The inconsistency between our results and those of previous studies may be due to the different tissue sources. The tissues used in the study reported by NEJM were formalin-fixed, paraffin-embedded HCC tissues, while frozen tissues were used in the current study. It would be worth conducting a multi-center study with a large sample size to help us obtain more stable experimental results.

Our study revealed that the Secreted Phosphoprotein 1 (SPP1) gene is a risk factor in the progression of HCC. Its mRNA level was higher in hepatoma cells than in normal hepatocytes, consistent with the previous research findings [31,32,33]. SPP1 is a multifunctional glycoprotein expressed in the macrophages across different tissues and is involved in biological processes such as inflammation, immune response, macrophage migration, tumor proliferation, and invasion [34,35]. A recent study discovered that SPP1 was an immune-related predictor of poor survival in patients with HCC. It was also found to mediate the interaction between HCC cells and macrophages through SPP1-CD44 and SPP1-PTGER4 interactions [36]. Spatial transcriptomics and scRNA-seq analysis revealed that SPP1^+^ macrophages and cancer-associated fibroblasts interacted to form an immune barrier near the HCC tumor boundary, blocking the infiltration of cytotoxic T lymphocytes into the tumor core, thereby inhibiting the therapeutic effect of immune checkpoint blockade [37]. Blocking the expression of SPP1 in mice could enhance the efficacy of immunotherapy [37], suggesting that SPP1 may serve as a potential target in HCC immunotherapy.

Previous studies have suggested that other prognostic factors, such as leukocyte cell-derived chemotaxin-2 (LECT2) and PON1, are also closely related to the biology of HCC. Xu et al. found that LECT2 can act as a novel and direct biomarker to estimate liver fibrosis in patients with chronic Hepatitis B [38]. Chi-Kuan et al. revealed that LECT2 antagonizes activation of MET receptor by recruiting the protein tyrosine phosphatase 1B to inhibit the vascular invasion of HCC. Furthermore, Antoine and his colleagues revealed that LECT2 could constrain the growth and progression of HCC through controlling the inflammatory monocytes [39]. Our research found that LECT2 is a protective factor for HCC, which is consistent with the previous conclusion that LECT2 acts as a tumor suppressor. Additionally, a recent study found that PON1 combined with three endoplasmic reticulum stress-related genes, AGR2, SSR2, and TMCC1, could accurately predict the survival outcomes in HCC patients [40]. Another study indicated that ANXA10 was poorly expressed in HCC tissues and cells, and in vivo studies have confirmed that the miR-513c/Cul4A/ANXA10 axis mediates lncRNA DLX6-AS1 to promote the progression of HCC [41]. In brief, these genes are promising as prognostic targets for HCC, and further exploration of mechanism will help us understand the biological processes of HCC.

Alpha-fetoprotein (AFP) is a glycoprotein that is mainly used as a serum marker for HCC in clinical diagnosis and efficacy monitoring. Studies have shown that serum AFP levels higher than 400 ng/mL suggest poor survival after a hepatectomy in patients with HBV-associated HCC [42]. However, AFP is not a strong prognostic marker because of its low sensitivity and specificity. Studies have shown that Golgi protein-73 (GP73), a resident Golgi glycoprotein, can serve as a new serum marker for HCC [43]. In addition, PIVKA-II was identified as a potential biomarker to complement AFP in the diagnosis of hepatocellular carcinoma [44]. A previous study compared the efficacy of four serum markers in monitoring HCC treatment response and found that des-γ-carboxy prothrombin was a more effective tumor marker than AFP and AFP-L3 [45]. AFP-L3 showed comparable accuracy to AFP, but no benefit was found in their combination, while GP73 did not play an important prognostic role. Moreover, in a systematic review of 112 studies on the accuracy of liquid biopsy analyses, researchers found that circulating tumor cells and cell-free DNA detection may help to determine the prognosis of patients and monitor HCC [46]. The 2022 updated Barcelona Clinic Liver Cancer strategy for prognosis prediction and treatment recommendation for HCC suggested evaluating prognosis according to the tumor burden and cancer-related symptoms, and defined by the AFP, albumin-bilirubin score, Child-Pugh, and MELD scores [47]. Although the risk score proposed in this study is based on mRNA expression levels in liver tissue rather than non-invasive biomarkers, it is an innovative exploration. Furthermore, it would be worth investigating if the predictive power of the risk score improves if combined with the previously established strategies.

In the present study, we established a risk model with good efficiency to predict the survival rate of patients with HCC. We evaluated the biological pathways, immune scores, immunotherapy responses, and drug susceptibility in high-risk and low-risk groups. Low-risk patients were more likely to benefit from immune treatment. In the past few years, immunotherapy has demonstrated strong anti-tumor activity in diverse malignant tumor types and has become a paradigm shift in cancer therapy [48,49,50]. About half of the patients with HCC receive systemic treatment, with the traditional first-line treatment being sorafenib or lenvatinib, and the second-line treatment being regorafenib, ramucirumab, or cabozantinib [51,52,53]. Currently, approximately 30 Phase III clinical trials are testing the efficacy of immunotherapy in all stages of HCC, which may significantly alter the management of HCC [54].

There are still many limitations in our study. First, we have not verified the robustness of the risk model in the local HCC cohort. Second, the molecular mechanism and functional verification of the prognostic genes in the development of HCC need further exploration in vivo and in vitro. Third, it will be necessary to compare the value of the existing biomarkers with the risk score based on bile acid and bile salt metabolism-related genes for the prognosis of HCC in a prospective cohort.

## 4. Materials and Methods

### 4.1. Research Subjects and Data Acquisition

TCGA is the world’s largest tumor gene database, collecting data from more than 20,000 samples of 33 types of cancer, including mRNA expression data, genomic variation data, methylation data, clinical data, etc. The mRNA expression data and clinical follow-up information of 365 patients with HCC were obtained from the TCGA database [55]. The Hepatocellular Carcinoma Database is a freely accessible database which enables a comprehensive understanding of gene expression patterns and multidimensional annotation of the transcriptomes in HCC [56]. As validation cohorts, the RNA-seq profiles and clinical records from 203 patients with HCC of the HCCDB18 dataset were retrieved from HCCDB.

GEO database includes high-throughput gene expression data submitted by research institutions around the world, which was created and maintained by The National Center for Biotechnology Information [57]. The GSE14520 (including 221 samples) and GSE76427 (including 115 samples) datasets of clinical survival data and mRNA expression data, as well as the GSE91061 (including 115 samples) and GSE135222 (including 27 samples) datasets containing immunotherapy information, were extracted from the GEO database. The information on the immunotherapy cohort IMvigor210 (including 298 samples) was provided by the IMvigor210CoreBiologies package [17].

### 4.2. Data Pre-Processing

Patients with incomplete survival status and follow-up data were excluded from further analysis. The transcriptome data of all genes in each sample were downloaded as FPKM (fragments per kilobase per million) for analysis. The DEGs were filtrated by limma package, and the screening threshold was set to | log_2_ (Fold change) | > 1 and false discovery rate < 0.05. Additionally, LASSO regression analysis was performed using the glmnet package to diminish the number of genes in the risk model [58].

### 4.3. Source of Bile Acid and Bile Salt Metabolism-Related Genes

The bile acid and bile salt metabolism-related genes involved in the pathway “bile acid and bile salt metabolism” were acquired from the MSigDB [59,60]. The list of the genes is detailed in Table S1. Additionally, univariate Cox regression analysis was conducted to determine the bile acid and bile salt metabolism-related genes associated with the prognosis of HCC.

### 4.4. Establishment of Clinical RiskScore Model

The following algorithm was used to evaluate the RiskScore of each patient: RiskScore = Σβi × Expi [61,62], where i represents prognostic genes, Exp represents the mRNA expression level, and β is the Cox regression coefficient. The Survminer package was applied to identify the optimal cutoff. Patients were assigned to the high-RiskScore or low-RiskScore groups according to the cutoff value. The Kaplan-Meier method was used to performed the survival analysis, and the log-rank test was used to compare the differences. The TimeROC package was applied to analyze the ROC, for which the efficacy was evaluated via the area under the ROC curve.

### 4.5. Evaluation of Immune State and Immunotherapeutic Responses

ssGSEA is an extension of the GSEA. ssGSEA allows the definition of an enrichment score, which represents the absolute degree of enrichment of each gene set in a given dataset [63]. ssGSEA was performed using the “GSVA” package to explore the immune score and biological pathways relevant to the immune system [64,65]. For each tumor sample, ESTIMATE calculates the Stromal score and Immune score of the sample based on the ssGSEA algorithm to evaluate the composition of stromal cells and immune cells. The synthesis of the Stromal score and the Immune score can further obtain the Estimate socre. The ESTIMATE package was used to calculate the proportion of stromal cells and immune cells in tumor samples [66]. TIDE is a computational framework for assessing the possibility of tumor immune escape in the gene expression profile of tumor samples. In our study, the TIDE algorithm was used to evaluate the immunotherapeutic response of the low-risk and high-risk groups [67].

### 4.6. Statistical Analysis

All statistical analyses were performed using GraphPad Prism (version 9.0) and R software (version 4.2.1). A two-tailed Student’s t-test was applied to analyze the continuous variables. *p*-values less than 0.05 were considered statistically significant.

## 5. Conclusions

In summary, our study determined two molecular subtypes with different survival probabilities and immune microenvironment characteristics based on bile acid and bile salt metabolism-related genes. Moreover, a seven-gene risk model could effectively predict the prognosis of patients with HCC and their immunotherapeutic response, which may contribute to better management of HCC.

## Data Availability

The data involved in this study are available from corresponding author based on rational requirement.

## Supplementary Materials

The following supporting information can be downloaded at: https://www.mdpi.com/article/10.3390/ijms24108597/s1.
