# Supplementary material for: Establishment and Validation of a Novel Risk Score for Hepatocellular Carcinoma Based on Bile Acid and Bile Salt Metabolism-Related Genes
Source: Int J Mol Sci. 2023 May 11;24(10):8597. doi: 10.3390/ijms24108597 (PMC10217959; doi:10.3390/ijms24108597)
Supplement: Supplementary file 1 [file ijms-24-08597-s001.zip › ijms-2283527-supplementary.pdf]

Table S1. The gene set of 45 bile acid and bile salt metabolism-related genes

No.	Gene Name	No.	Gene Name
1	ABCB11	24	NCOA2
2	ABCC3	25	NR1H4
3	ACOT8	26	OSBP
4	ACOX2	27	OSBPL1A
5	AKR1C1	28	OSBPL2
6	AKR1C2	29	OSBPL3
7	AKR1C3	30	OSBPL6
8	AKR1C4	31	OSBPL7
9	AKR1D1	32	OSBPL9
10	ALB	33	PTGIS
11	AMACR	34	RXRA
12	BAAT	35	SCP2
13	CH25H	36	SLC10A1
14	CYP27A1	37	SLC10A2
15	CYP39A1	38	SLC27A2
16	CYP46A1	39	SLC27A5
17	CYP7A1	40	SLC51A
18	CYP7B1	41	SLC51B
19	CYP8B1	42	SLCO1A2
20	FABP6	43	SLCO1B1
21	HSD17B4	44	SLCO1B3
22	HSD3B7	45	STARD5
23	NCOA1		

Note: The gene set was downloaded from the following site: http://www.gsea-msigdb.org/gsea/msigdb/human/geneset/REACTOME_BILE_ACID_AND_BILE_SALT_METABOLISM.html?keywords=metabolis
